# Recent Advances in Nanotechnology‐Mediated Noninvasive Transdermal and Topical Delivery of Proteins

**DOI:** 10.1002/smsc.202400175

**Published:** 2024-07-16

**Authors:** Junghyeon Ko, Jeong‐Uk Kim, Subin Choi, Ye‐Sol Kim, Su‐Bin Park, Joo‐Young Kim, Hyeon‐Jin Kim, Young‐Sun Lee, Young‐Hyeon An, Nathaniel S. Hwang

**Affiliations:** ^1^ School of Chemical and Biological Engineering Institute of Chemical Processes Seoul National University Seoul 08826 Republic of Korea; ^2^ Interdisciplinary Program in Bioengineering Seoul National University Seoul 08826 Republic of Korea; ^3^ BioMAX/N‐Bio Institute Institute of Bio‐Engineering Seoul National University Seoul 08826 Republic of Korea; ^4^ Institute of Engineering Research Seoul National University Seoul 08826 Republic of Korea

**Keywords:** drug deliveries, nanoparticles, proteins, topical deliveries, transdermal deliveries

## Abstract

Protein therapeutics are emerging as essential technologies due to their functional and chemical properties. However, their application is currently limited to delivery by oral and injection methods—the former being inefficient and the latter invasive and potentially tissue damaging. Researchers are, therefore, exploring noninvasive delivery systems for proteins through the skin, including transdermal and topical delivery. The large molecular size of proteins presents a key challenge for skin penetration, hindering their ability to penetrate the dense skin lamellar structure. This review focuses on using nanoparticles as carriers to increase protein stability and enhance skin penetration. The use of noninvasive or minimally invasive enhancers for controlling and improving penetration depth is also examined. Furthermore, the physical properties of nanoparticles that affect delivery are evaluated, aiming to propose ways to advance transdermal and topical delivery methods in the future.

## Graphical abstract

Protein transdermal and topical delivery face significant hurdles due to the large size of proteins and the dense skin layer. Nanoparticle‐guided delivery is an emerging technology designed to increase protein stability and skin penetration. The control and enhancement of penetration by combining enhancers with nanoparticles have been analyzed. Moreover, the physical properties of nanoparticles that affect delivery have been examined.

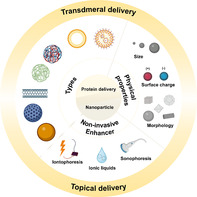

## 1 Introduction

In recent years, the demand for effective and noninvasive drug delivery systems has significantly increased.^[^ ^1^ ^]^ Traditional methods, such as oral and injection‐based delivery, have various drawbacks that limit their use. For instance, oral delivery exhibits low efficiency due to the harsh conditions of the gastrointestinal tract and existing physical barriers.^[^ ^2^ ^]^ On the other hand, injection‐based delivery shows high efficacy as it directs drugs to the targeted areas. However, these invasive methods induce pain and pose challenges for individuals with a needle phobia. Furthermore, some drugs, such as insulin, require multiple doses throughout the day, making it inconvenient and disruptive to patients’ everyday routines.^[^ ^3^ ^]^ Therefore, numerous techniques have been developed to deliver drugs directly to systemic circulation via noninvasive systems.^[^ ^4^ ^]^

Transdermal and topical delivery systems have emerged as alternative drug delivery methods. These systems provide several advantages, including noninvasiveness, improved patient compliance, and reduced side effects, such as minimizing the loss of activity caused by degradation or denaturation during oral delivery. As a result, drug‐delivering patches for small drugs like Asenapine, Buprenorphine, and Estrogen have been developed.^[^ ^5^ ^]^ However, the delivery of protein therapeutics presents a challenge due to their large size. Several other challenges persist beyond the size of proteins, including low solubility, distribution, and stability.^[^ ^6^ ^]^ Maintaining their activity is challenging as multiple proteinases can degrade these proteins.^[^ ^6^, ^7^ ^]^ Additionally, there are various purposes for delivering proteins, including controlled release inside the dermal layer, targeting topical delivery, transdermal delivery for systemic circulation, etc.^[^ ^8^ ^]^

The stratum corneum, the outermost layer of the skin, is composed of corneocytes and lipids that form a lamellar structure.^[^ ^9^ ^]^ This dense barrier protects the body from hazardous stimuli, including chemical, biological, and electromagnetic damage.^[^ ^10^ ^]^ Additionally, the hydrophobicity of the skin hinders the permeation of hydrophilic materials. Therefore, many molecules over 500 Da hardly pass through the stratum corneum. To overcome these challenges, researchers have focused on developing nanocarriers and enhancers to improve the delivery efficacy of protein therapeutics. Various noninvasive delivery methods, such as ultrasound‐assisted delivery, electroporation, and the topical application of enhancers, have been studied to enhance the permeation of macromolecules such as peptides, proteins, and nucleic acids through the skin.^[^ ^10^, ^11^ ^]^ Nanocarriers or nanoparticles are known to enhance the stability and solubility of proteins while protecting the drugs, and they increase penetration depth while maintaining the activities of proteins.^[^ ^12^ ^]^ Nanotechnology could also be used for diagnostic purposes such as cell targeting, intracellular tracking, or analyzing drug delivery profiles when delivering through the skin, thereby increasing the potential to understand the pathology of drug delivery.^[^ ^13^ ^]^

**Figure 1:**
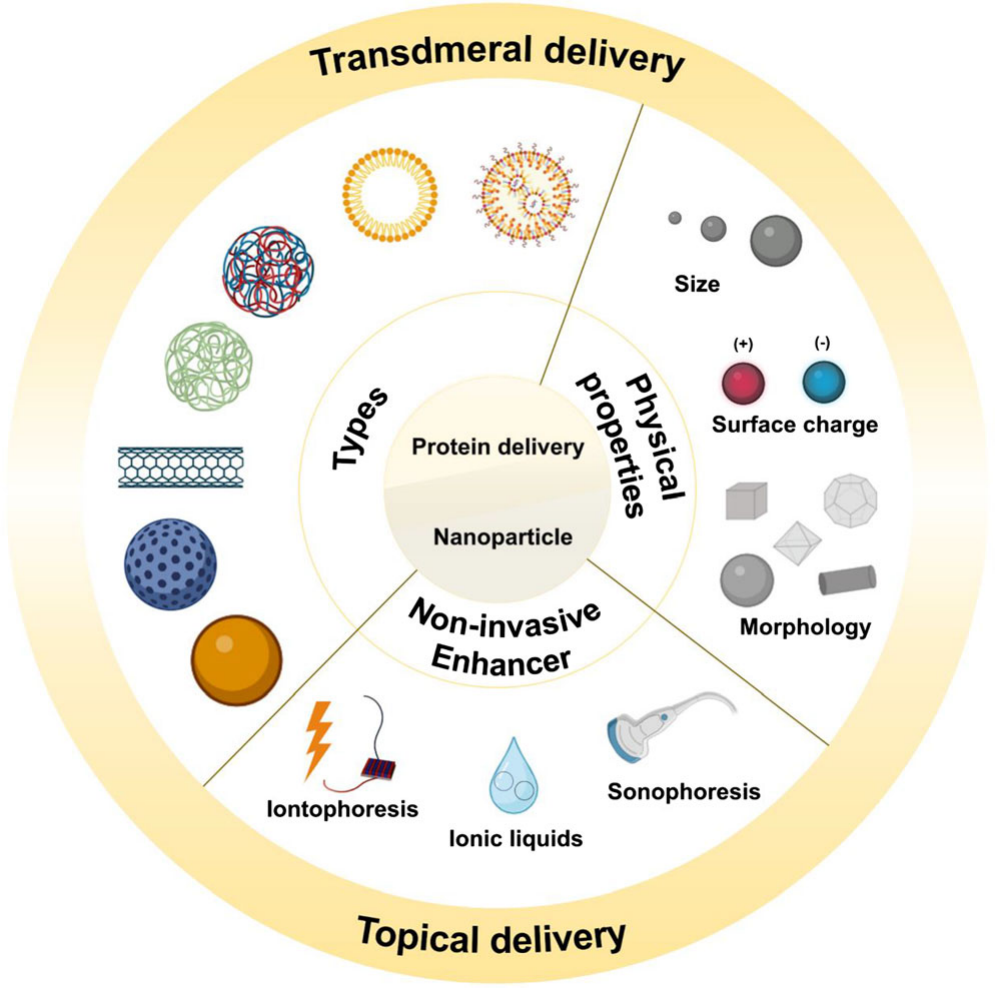
Overview of nanoparticles for protein transdermal and topical delivery, including physical properties affecting delivery efficiency and enhancers to control the delivery. The figure was created with Biorender.com.

Nanoparticles come in various types depending on the materials used in their fabrication, including lipid‐based particles (liposomes, microemulsions, lipid nanoparticles (LNPs), etc.), polymeric nanoparticles, inorganic nanoparticles, and carbon nanotubes. These types of particles exhibit different mechanical, chemical, and physiological properties. Moreover, their properties, such as surface charge, surface area, and hydrophobicity, can be tailored by modifying the particle composition or surface depending on the types of proteins, their quantities, and target locations. Moreover, certain properties like surface charge and morphology can be effectively combined in a system with enhancers, which are carefully chosen considering the physical and chemical properties of the skin, such as charge, pH, lipophilicity, and density. Enhancers such as iontophoresis, ionic liquids (IL), and microneedles are examples that can increase and maximize the advantages of utilizing nanoparticles (**Figure** **1**).

This review explores recent advances in nanotechnology‐based, noninvasive transdermal and topical delivery for protein, along with their potential applications. We will discuss the properties affecting skin penetration and protein delivery by comparing the properties of nanoparticles. This article aims to provide insights into the research conducted thus far in this field and identify areas that warrant further study.

## 2 Nanoparticle‐Based Transdermal and Topical Delivery Platforms

Given that the skin is lipophilic and has a dense lamellar structure, the selection of materials used or the surface modification is crucial when designing skin‐penetrating nanocarriers. The base materials, such as lipids, polymers, or metallic compounds, along with the nanoparticle fabrication method, determine the nanoparticles’ types. Different types of nanoparticles are selected based on various needs such as stability, flexibility, encapsulation efficiency, and release ratio, which in turn affect the delivery dose, penetration depth, and targeting efficiency. Moreover, penetration mechanisms, which vary depending on the type, have been studied because maintaining the stability of particles and drugs while passing through the stratum corneum is a significant issue.^[^ ^14^ ^]^ Therefore, recent studies on protein transdermal or topical delivery have focused on exploring the impacts of different types of nanoparticles.

### 2.1 Lipid‐Based Nanoparticle‐Based Delivery Systems

Lipid‐based nanoparticles have been studied for use in transdermal and topical delivery systems because their lipophilic properties show affinity with the outermost layer of the skin, making it easier to penetrate the stratum corneum (**Table** **1**).^[^ ^15^ ^]^ Depending on the types and amounts of lipids and solvents utilized, there are many types of lipid‐based nanoparticles, such as liposomes, LNPs, microemulsions, and so on. Therefore, studies on the transdermal and topical delivery of small drugs using lipid‐based nanoparticles, such as liposomes,^[^ ^16^ ^]^ transfersome,^[^ ^17^ ^]^ ethosome,^[^ ^18^ ^]^ and emulsions,^[^ ^19^ ^]^ have been reported.

**Table 1:** Lipid‐based nanoparticles‐assisted protein transdermal and topical delivery.

Nanoparticle type	Subtype	Characteristic (size, zeta potential)	Protein	Enhancer	Results	Reference
Lipid‐based nanoparticle	Liposome	134.8 ± 0.6 nm	Tyrosinase (PaTy)	Microneedle roller	Photoactivatable tyrosinase (PaTy) was loaded onto liposomes to increase stability and improve topical delivery efficiency. PaTy was successfully delivered to ex vivo porcine skin and in vivo mouse skin, then activated by UV light to synthesize melanin within the tissue. The synthesized melanin protected the skin from UV radiation, mimicking the natural biosynthesis system of melanin and melanosomes.	[27a]
		−34.2 ± 2.7 mV				
	Liposome	84.96 ± 0.45 nm	IL‐4	–	Gene therapy for psoriasis was demonstrated.	[27b]
		50.8 ± 2.23 mV				
	Liposome	151.9 – ≈158.4 nm	Collagen	–	Penetration of artificial 3D skin model	[27g]
		−24.3 – ≈19.2 mV				
	Liposome	–	INFα2b	–	Working as an antifibrogenic agent in dermal wounds in the guinea pig	[27d]
		–				
	Liposome	126.6 nm	BSA, adipose‐derived stem cell (ADSC) secretome	Iontophoresis (RED)	BSA‐encapsulated liposomes delivered up to 393.88 ± 33.29 μm without RED and 506.67 ± 51.07 μm with RED through ex vivo porcine skin. ADSC secretome‐encapsulated liposomes were delivered to mouse skin in vivo, preventing and alleviating photoaged skin by stimulating elastin and HA synthesis.	[27e]
		−25.8 mV				
	Liposome w. silver nanoparticle	28 ± 2 nm	OVA	Iontophoresis	Higher OVA skin penetration was observed with silver nanoparticles than with carrier systems without silver nanoparticles.	[27f]
		−14 ± 1 mV				
	Ethosome (hyaluronic acid (HA) and galactosylated chitosan (GC) modiﬁed)	Approximately 200 nm	OVA	–	Transdermal vaccination and antitumor immunity were observed.	[27c]
		38.0 ± 2.11 mV				
	Transfersome	241.33 ± 17, 171 ± 12.12 nm	hGH	–	The transdermal flux of hGH‐loaded transfersome was measured as 11.16 and 9.77 ng cm^−2^ h^−1^	[27h]
		−23.67 ± 4.7, −21 ± 3 mV				
	Emulsion (gel‐in‐oil)	–	GF cocktail (VEGF, EGF, bFGF, TGF‐1, IGF‐α)	–	Prevention and repair of UV B‐induced skin damage such as erythema, skin moisture reduction, inflammation, and skin thickness were observed.	[29]
		–				
	Emulsion (gel‐in‐oil)	<250 nm	EGFP, bFGF	–	EGFP was delivered up to 190 μm into the skin in vitro. A higher amount of hemoglobin was confirmed with bFGF delivery in mouse back skin in vivo, compared to the G/O and Hep‐G/O conditions.	[31a]
		–				
	Emulsion (gel‐in‐oil)	–	bFGF	–	Subcutaneous angiogenesis was induced.	[31b]
		–				
	Emulsion (IL‐in‐oil)	16.3 nm, 19.6 nm	Insulin	–	In vitro transdermal flux with three different types of microemulsion was observed to be 0.35 ± 0.07 × 10^−2^, 1.96 ± 0.30 × 10^−2^, and 2.68 ± 0.40 × 10^−2^ μg cm^−2^ h^−1^, while the passive delivery of insulin showed a flux of 0.08 ± 0.02 μg cm^−2^ h^−1^. Furthermore, a blood glucose‐lowering effect to under 150 mg dL^−1^ was observed for 48 h in STZ‐induced diabetic mice treated with the microemulsion.	[80a]
		–				
	Emulsion (water‐in‐oil)	41.05 ± 8 nm	Insulin	–	Transdermal flux was observed as 1.75 μg cm^−2^ h^−1^ in the Strat‐M membrane, a surrogate of human skin.	[88]
		–				
	Emulsion (solid‐in‐oil)	129.7 nm	BSA	–	Transdermal delivery of emulsion was observed for 72 h in pig ear skin in vitro, and a cumulative amount increase was observed as the emulsion solution concentration increased.	[31c]
		–				
	Emulsion (solid‐in‐oil)	257 nm, 236 nm, 214 nm	Insulin, EGFP, HRP	–	After 48 h, 1.02 ± 0.24 μg cm^−^^2^ of insulin penetrated the skin layers, including the stratum corneum (SC), epidermis, and dermis, when treated with the S/O nanodispersion. In contrast, passive treatment resulted in only 0.14 ± 0.08 μg cm^−^^2^ of insulin penetration. EGFP and HRP penetrated the SC to a depth of about 15 μm.	[30]
		–				
	Lipid‐based IL nanocarrier	279 ± 61 nm	OVA	–	Transdermal flux was observed as 7.6 ± 0.6 μg cm^−2^ h^−1^ in skin in vitro. Transdermal vaccination and antitumor immunity were observed.	[80b]

Liposomes are bilayer structures of phospholipids formulated spontaneously in an aqueous environment. Since the hydrophobic tails and the hydrophilic heads of phospholipids create regions that can encapsulate both hydrophobic and hydrophilic drugs, they have been widely used as drug carriers.[^16^, ^20^] Therefore, liposomes encapsulating proteins, which are either hydrophobic or hydrophilic macromolecules, have also been studied for a long time.^[^ ^21^ ^]^ To overcome the limitations of conventional liposomes, such as poor skin penetration efficiency and unstable membranes, which can result in a short half life, innovative designs of liposomes have been suggested. Among these designs of liposomes are transfersomes, ethosomes, niosomes, and others. Gregor Cevc suggested the transfersomes, which consist of phospholipids and membrane softening agents (such as surfactants Tween 80, Tween 20, Span 80, and sodium cholate).^[^ ^22^ ^]^ The softening agents increase deformability and penetration efficiency through narrow pores. Consequently, many transdermal and topical delivery studies have been conducted using transfersomes to penetrate the narrow structure of the stratum corneum.^[^ ^23^ ^]^ In addition, ethosomes are elastic nanoparticles developed from liposomes, containing a high ethanol content as a permeation enhancer.^[23b]^

Research suggests that the skin penetration mechanism of lipid‐based nanoparticles is induced by the deformation of particles while passing through the lamellar structure of the skin. Therefore, the stability of lipid‐based nanoparticles during skin penetration is controversial. Some studies suggest that, without any enhancer, conventional lipid‐based particles such as liposomes or emulsions break down during the penetration of the epidermis.^[^ ^14^ ^]^ Many modifications, such as designing transfersomes, ethosomes, or nanoemulsions, have been introduced to enhance stability or flexibility.[^17^, ^24^] As flexibility increases, the structure of the particle elastically deforms during penetration and safely passes through the lamellar structure of the skin.^[^ ^25^ ^]^ Chang Yang et al. introduced a simulation study of transfersome passing through lipid layers, demonstrating its high deformability.^[^ ^26^ ^]^ Lipid‐based nanoparticles have been extensively researched compared to other particles, making them promising carriers for applications in protein delivery.^[^ ^27^ ^]^

**Figure 2:**
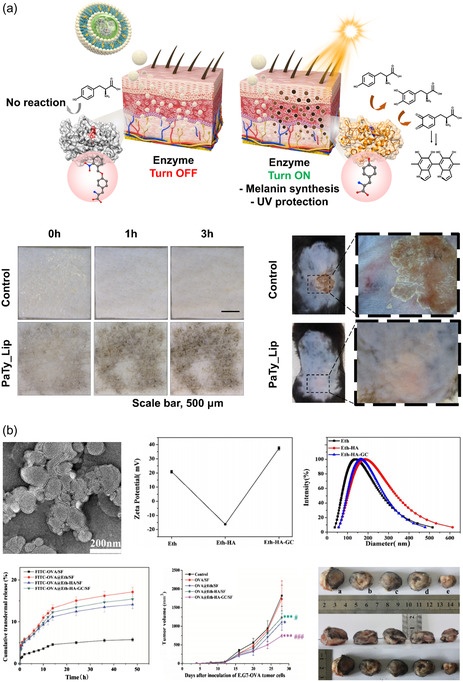
Liposome and modified liposome‐assisted protein delivery. a) Photoactivatable tyrosinase (PaTy) was encapsulated in liposome nanoparticles to increase the stability of the enzyme and skin permeability. Tyrosinase was successfully activated within ex vivo porcine skin, leading to melanin synthesis. The melanin synthesized in in vivo mice protected their skin from UV radiation and reduced the formation of scabs. Reproduced with Permission.^[27a]^ Copyright 2022, Wiley. b) OVA loaded into ethosomes was fabricated with hyaluronic acid and galactosylated chitosan modifications. This system stimulated the immune response and inhibited the growth of EG7 tumors. Reproduced with Permission.^[27c]^ Copyright 2020, Elsevier.

Uk‐Jae Lee et al. designed an artificial melanosome by encapsulating photoactivable tyrosinase (PaTy) in liposomes (**Figure** **2**a).^[27a]^ The size and the zeta potential of the particle are 134.8 ± 10.6 nm and −34.2 ± 2.7 mV, respectively. Topically delivered tyrosinase could be activated by light to synthesize melanin by reacting with endogenous L‐tyrosine, thereby protecting skin tissues from UV radiation with synthesized artificial melanin. They observed the activation of delivered PaTy in ex vivo porcine skin and in vivo C57BL/6 mice. Synthesized melanin protects the skin against UV irradiation, as demonstrated by less scab formation and thinner skin.^[27a]^

Ethosomes, a type of decorative liposomes, were used for ovalbumin (OVA) skin delivery for vaccination by Xingxing Yang et al. (Figure 2b).^[27c]^ Ethosomes were coated with hyaluronic acid (HA) and galactosylated chitosan (GC). HA was used to improve stability and transdermal efficiency, while GC increased cell adhesion and targeted dendritic cells. OVA‐loaded ethosomes showed a size under 150 nm, which increased to over 150 nm after coating. Zeta potential remained positive before and after coating, showing + 20.9 ± 0.95 mV and + 38.0 ± 2.11 mV, respectively. Ethosomes were loaded onto silk fibroin nanofiber‐based patches for controlled release. Transdermal efficiency was observed using Franz diffusion cell in vitro with mouse skin and in vivo with C57BL/6 mice. Transdermal immunization by OVA‐loaded ethosome carriers showed a decrease in tumor volume, with an even higher decrease observed with GC and HA‐coated ethosome carriers.^[27c]^

Although some studies demonstrate the therapeutic effect or delivery efficacy of conventional liposomes (Table 1), low stability and low flexibility are significant drawbacks.^[^ ^14^ ^]^ Therefore, enhancers or customizations like transfersomes, ethosomes, or emulsions are needed to improve skin penetration. Moreover, polymer coating or additional surface modifications for skin affinity or skin opening could increase transdermal efficacy or cellular uptake. However, studies such as those by Xinxing Yang et al. demonstrate that polymer coating can alter the physical properties of the particles, such as increased size or surface charge change, potentially decreasing transdermal delivery.^[27c]^ Therefore, this indicates that not only the types or surfaces of nanoparticle materials are important in transdermal delivery, but also their physical properties must be considered simultaneously.

Emulsions or microemulsions are uprising ideas for delivering drugs transdermally due to their high portions of lipid phases, which increases transdermal efficiency.^[^ ^28^ ^]^ Emulsions are well‐known systems that use oil and water phases to load and deliver hydrophobic or hydrophilic materials by forming single emulsions (water‐in‐oil or oil‐in‐water emulsions). Additionally, by creating double emulsions (water‐in‐oil‐in‐water or oil‐in‐water‐in‐oil emulsions), they can deliver amphiphilic materials or both hydrophobic and hydrophilic materials together, thus increasing loading efficiency.^[19a]^ Moreover, their flexibility helps to improve the penetration of the stratum corneum. Therefore, proteins could be protected by a lipid layer or oil phase and delivered efficiently.

**Figure 3:**
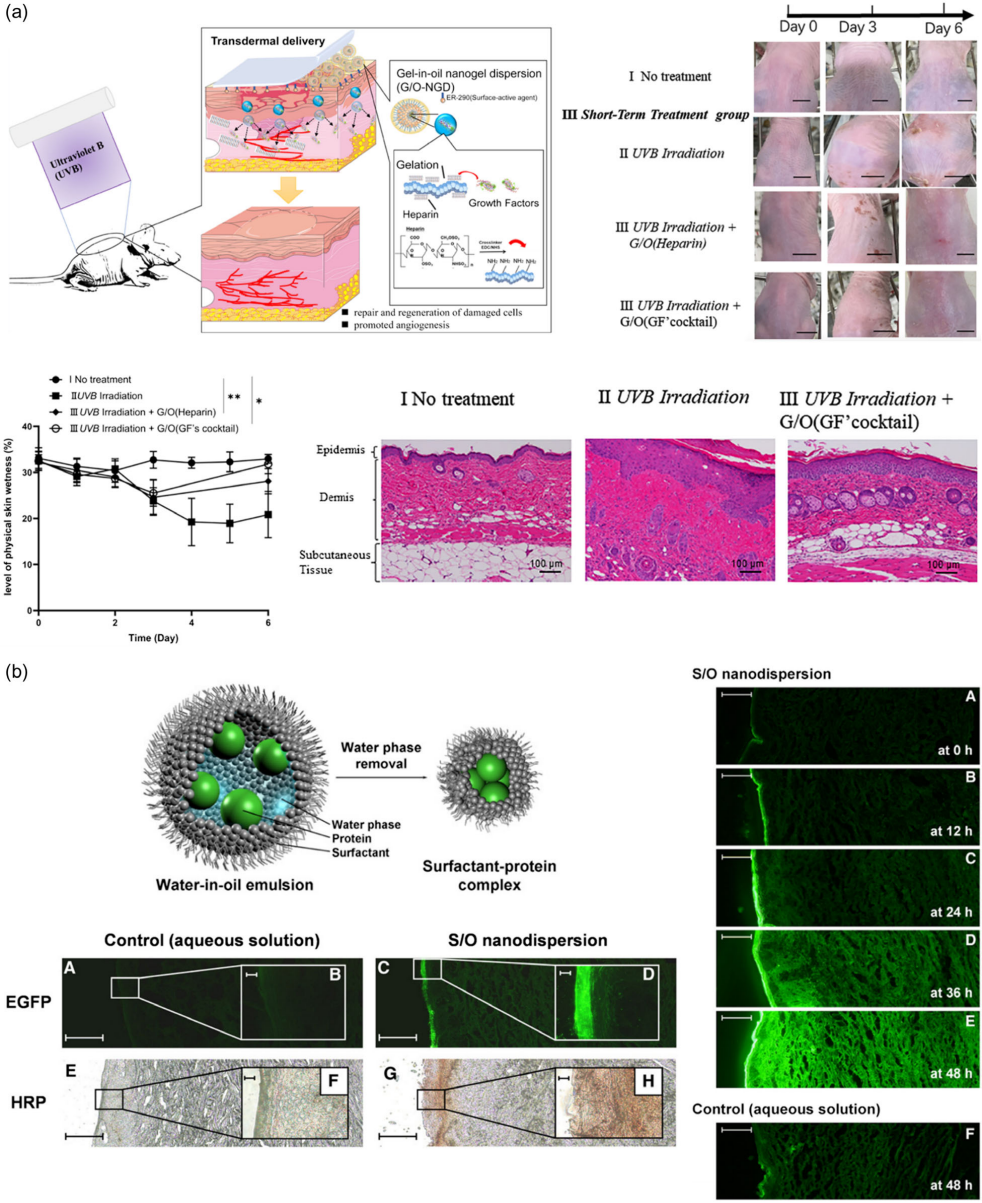
Lipid‐based nanoparticle‐assisted protein delivery. a) GFs were loaded into gel‐in‐oil emulsion nanoparticles. The delivered GFs reduced UV‐induced skin damage, including water loss and dermal thickening. Reproduced with Permission.^[^ ^29^ ^]^ Copyright 2023, ACS Publications. b) The solid‐in‐oil emulsion system was designed to deliver proteins such as insulin, EGFP, and HRP. Particles loaded with insulin penetrated the dermal layer, while particles loaded with EGFP and HRP were delivered to the epidermis, exhibiting fluorescence and catalytic activity. Reproduced with Permission.^[^ ^30^ ^]^ Copyright 2008, Elsevier.

Yi Zhang et al. developed a gel‐in‐oil nanoemulsion system for delivering a growth factor (GF) cocktail (VEGF, EGF, bFGF, TGF‐1, IGF‐α) to prevent and reduce UVB‐induced damage (**Figure** **3**a).^[^ ^29^ ^]^ EDC/NHS‐coupled heparin and gelatin were used for the aqueous phase and further gelation in 4 °C condition. Heparin increased the encapsulation efficiency of GFs due to its high affinity for binding to GFs. Isopropyl myristate (IPM) and sucrose erucic acid ester (ER‐290) were used for the oil phase. The emulsion size was optimized to be under 250 nm, and it was topically delivered to skin cells effectively, showing reduced UV‐induced damage.^[^ ^29^ ^]^

Yoshiro Tahara et al. removed the water phase to create a surfactant (ER‐290)–protein complex system (solid‐in‐oil nanodispersion), which can load different proteins such as insulin, enhanced green fluorescent protein (EGFP), and horseradish peroxidase (HRP) (Figure 3b).^[^ ^30^ ^]^ The average sizes measured were 257, 236, and 214 nm for the insulin, EGFP, and HRP‐encapsulated nanocarriers, respectively. The insulin‐encapsulated nanocarrier showed high penetration efficiency transdermally, passing through the dermal layer. Similarly, EGFP and HRP‐loaded nanodispersions also exhibited high penetration abilities, passing through the stratum corneum and epidermal layer.^[^ ^30^ ^]^ Their research also demonstrates deep penetration of insulin into the dermis with a nanocarrier, while HRP and EGFP accumulate on the epidermis. This indicates the impact of protein size or molecular weight on penetration. Comprehension of the correlation between protein size and skin penetration could further help in designing nanocarriers for protein skin delivery.

Likewise, emulsions have been extensively studied as transdermal delivery carriers due to their lipophilicity and easy modification by changing the ratio of reagents or solvents. Most emulsion‐based protein delivery systems demonstrate effective therapeutic effects without using any enhancers (Table 1). Although emulsions have high flexibility, conventional water‐in‐oil and oil‐in‐water emulsions show low stability due to phase separation over time. This issue has been addressed by designing solid‐in‐oil or gel‐in‐oil emulsions without another solvent, thereby eliminating the possibility of phase separation.^[^ ^29^, ^30^, ^31^ ^]^ However, further studies are needed on the amount and size of proteins loaded, as the lack of solvents indicates the properties of the proteins could affect the flexibility of the particles and their penetration efficiency.

LNPs, currently spotlighted due to their use as COVID‐19 vaccine carriers, are typically designed with four types of lipids, including ionizable lipids, helper lipids, cholesterol, and PEG‐lipids, and they are effective in protecting and delivering mRNA or proteins.^[^ ^32^ ^]^ However, they were only used via injection due to their low stability, limited penetration, and lag‐time delay, which are critical factors in transdermal or topical delivery.^[^ ^33^ ^]^ Jin Young Kim et al. developed a lipid‐based micelle‐assisted 5 kDa peptide topical delivery system called discoidal LNPs, which exhibited relatively high penetration efficiency, reaching depths of up to 36 μm.^[^ ^34^ ^]^ However, the carrier used in this study is closer to a micelle and has not used multiple types of lipids like mRNA vaccine carriers. Therefore, studies on delivering large‐molecular‐weight proteins of more than 10 kDa or using lipid types as mRNA‐carrying LNPs have not yet been reported. Increasing stability and flexibility by altering the composition of particles and using enhancers would enhance penetration ability.

### 2.2 Polymeric Nanoparticle‐Based Delivery Systems

Polymeric nanoparticles could be designed with various polymers, allowing for a wide range of properties (**Table** **2**).^[^ ^35^ ^]^ Typically, polymeric particles are very stable and tightly structured with flexibility, making it easy to control the release of drugs inside and ensuring stable prevention and delivery through the skin.^[^ ^36^ ^]^ Since the 1960s, research on using synthetic polymers as implantable carriers has been actively researched, and in the 1970s, protein‐release technology from sustained‐release drug delivery systems to polymeric carriers was established.^[^ ^37^ ^]^

**Table 2:** Polymeric nanoparticles‐assisted protein transdermal and topical delivery.

Nanoparticle type	Subtype	Characteristic (size, zeta potential)	Protein	Enhancer	Results	Reference
Polymeric Nanoparticle	Chitosan	258 ± 15 nm	OVA, gp100	–	Transdermal vaccination and antitumor immunity were observed.	[44b]
		27.6 ± 4.7 mV				
	Carboxymethyl chitosan	347.1 ± 3 nm	bFGF	Sonophoresis	With 120 mA applied current of sonophoresis, bFGF in nanocarrier delivered 7.57 ± 0.71 μg mL^−1^ while 5.10 ± 0.48 μg mL^−1^ were detected without nanocarrier.	[44a]
		–				
	Carboxymethyl chitosan (linoleic acid and arginine conjugated)	229.4 ± 1.83 nm	Insulin	Microneedle	Insulin‐loaded nanocarrier transdermal flux was measured as 11.05 ± 1.45 μg cm^−2^ h^−1^ without microneedle and 25.46 ± 0.58 μg cm^−2^ h^−1^ with microneedle. Low blood glucose level was maintained for 6 h in the STZ‐induced diabetic rat model.	[44d]
		–				
	PLGA (chitosan coated)	98.4 ± 36.8 nm	Hen egg‐white lysozyme	Iontophoresis	Transdermal vaccination. With the iontophoresis system, HEL‐specific IgG1 and IgG2a titers were 7.4 × 10^4^ and 6.6 × 10^2^ with the nanocarrier, while 4.0 × 10^4^ and 4.2 × 10 were observed without the carrier.	[44c]
		1.28 × 10^−2^ M (surface charge number)				
	Pluronic F127 (chitosan conjugated)	≈60 nm Chitosan conjugated: ≈70 nm	BSA, insulin	–	With the FITC‐conjugated protein, nanocarrier‐mediated delivery to human cadaver skin in vitro showed 22.3 ± 2.2 and 20.7 ± 3.6 relative fluorescent intensity for BSA and insulin compared to 2.0 ± 0.1 and 2.0 ± 0.1 without nanocarrier.	[44e]
		≈4 mV Chitosan conjugated: ≈12 mV				
	Pluronic F127	33 ± 8.73 nm	R848, OVA	Microneedle	Transdermal vaccination and antitumor immunity were observed.	[82b]
		–				
	Polypyrrole	423.5 nm	Insulin	Iontophoresis	Insulin flux was measured with Franz diffusion cell with rat skin. Passive delivery showed 0.36 ± 0.09 μg cm^−2^ h^−1^. Anionic iontophoresis showed 9.59 ± 2.65 μg cm^−2^ h^−1^, and Cationic iontophoresis showed 15.67 ± 4.24 μg cm^−2^ h^−1^.	[47]
		≈38 mV				
	Hyaluronic acid (1‐methyl‐DL‐tryptophan (1‐MT) conjugated)	151 nm	Anti‐PD1 antibody (aPD1)	Microneedle	Antitumor immunotherapies targeting immunoinhibitory receptor PD1 and immunosuppressive enzyme IDO were demonstrated.	[46]
		−17.1 ± 0.2 mV				
	Hyaluronic acid (2‐nitroimidazole conjugated)	–	Insulin, GOx	Microneedle	Duration of normal blood glucose level (<200 mg dL^−1^) was observed for about 4 h.	[82c]
		–				
	Poly(phenylboronic acid) (pillar[5]arene and a paraquat‐modiﬁed)	110 nm	Insulin, GOx	Microneedle	Duration of normal blood glucose level (<200 mg dL^−1^) was observed for about 5 h while injection of insulin was maintained for 2 h.	[82d]
		**–**				

Polymeric nanoparticles are typically classified into natural polymeric nanoparticles (such as chitosan and alginate) and synthetic polymeric nanoparticles (such as poly(lactic‐co‐glycolic acid) (PLGA), polylactides (PLA), and poly(ethyl methacrylate)), based on the type of polymer from which they are fabricated. Polymeric nanoparticles based on these polymers observe high encapsulation efficiency and biocompatibility.^[^ ^36^ ^]^ Different types of polymers have distinct advantages and skin penetration mechanisms. Chitosan is one of the widely used polymers as a nanocarrier due to its primary amine group, which can be easily functionalized with other groups, and its positive charge increases the attractive force with the negatively charged skin. Chitosan induces keratin conformation changes and transiently opens the tight junctions between epithelial cells, which help penetrate the skin.^[^ ^38^ ^]^ HA has been utilized in skin biomedical engineering since it has shown tissue regeneration, antiwrinkle, skin hydration, etc. For the skin penetration mechanism of HA, HA hydrates the stratum corneum and induces swelling of corneocytes, leading to ruptures between corneocytes. Therefore, these microstructural changes in the lipid layer allow the polymer to penetrate. Moreover, the HA receptor on the cell surface will enable HA to interact with dermal cells, and it is even used to target tumor cells that overexpress the HA receptor. However, their molecular weight limits their skin penetration ability, so only the low molecular weight is known to be suitable intradermally or transdermally.^[^ ^39^ ^]^ PLGA is also utilized commercially or in research due to its high biocompatibility, biodegradability, and drug stability. PLGA particles can load both hydrophilic and hydrophobic macromolecules or small molecules.^[^ ^40^ ^]^ Although some research suggests that PLGA particles have flexible properties, which can increase stratum corneum penetration efficacy, it remains controversial because other research suggests that they can only penetrate through hair follicles and accumulate. Therefore, modifying PLGA for transdermal delivery may be necessary.^[^ ^41^ ^]^ Polycaprolactone (PCL) is mainly used due to its biocompatibility and biodegradability properties.^[^ ^42^ ^]^ Moreover, PCL is a hydrophobic polymer, so most delivery systems combine hydrophilic materials to increase water solubility.^[^ ^43^ ^]^

Among the various polymers with different advantages, chitosan is the most studied polymer as a transdermal delivery carrier for proteins.^[^ ^44^ ^]^ Although studies on HA‐protein conjugates for transdermal delivery, with undefined sizes, have been conducted,^[^ ^45^ ^]^ there has been little research on transdermal delivery systems utilizing HA nanoparticles for protein encapsulation.^[^ ^46^ ^]^

**Figure 4:**
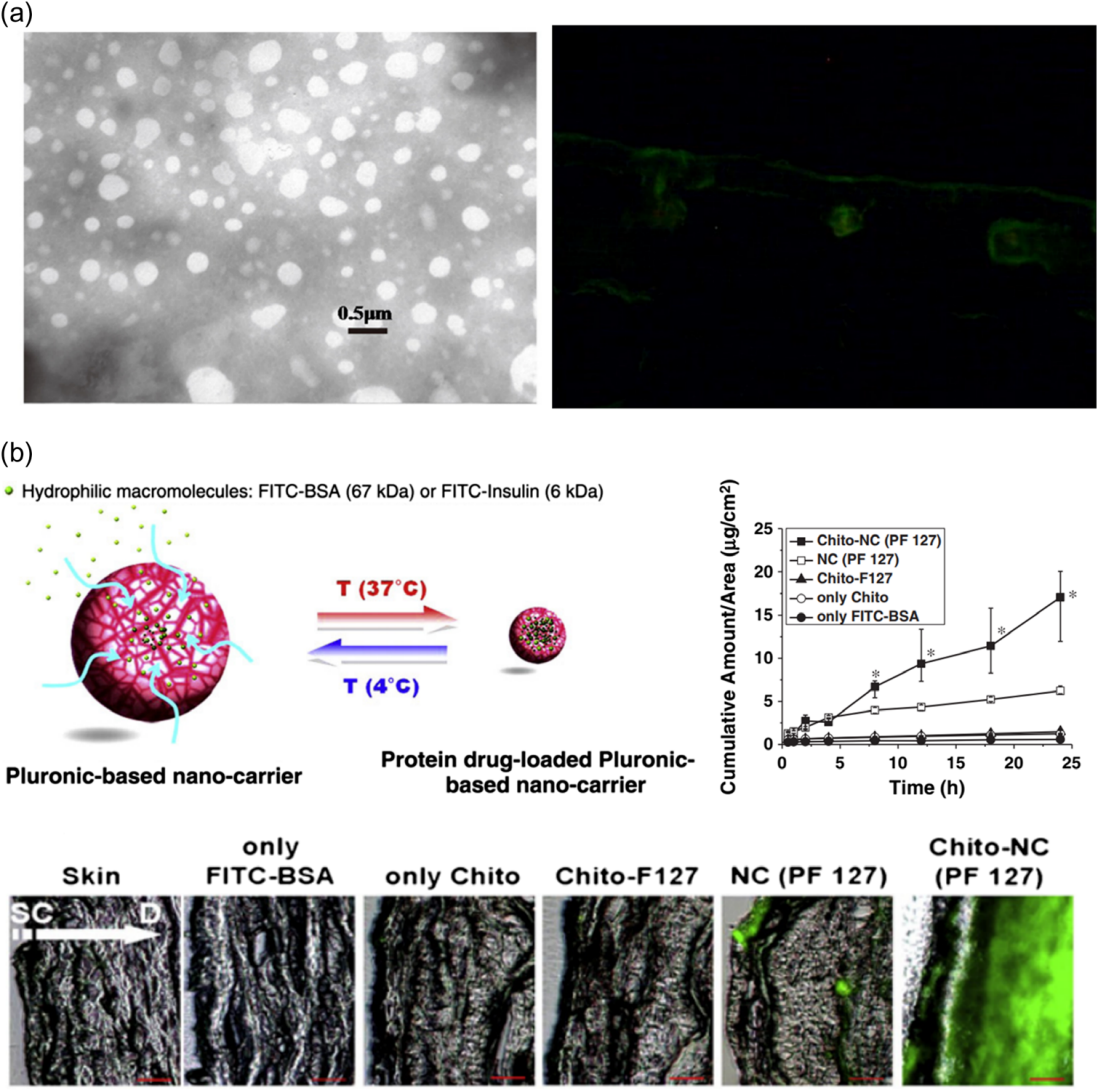
Polymeric nanoparticle‐assisted protein delivery. a) OVA was loaded into chitosan nanoparticles. In vivo studies in mouse skin showed that OVA had deeper penetration when delivered with nanoparticles than OVA alone. Reproduced with Permission.^[44b]^ Copyright 2014, Elsevier. b) Chitosan‐conjugated, pluronic F 127‐based nanocarrier was fabricated to deliver insulin and BSA into the skin. Reproduced with Permission.^[44e]^ Copyright 2011, Elsevier.

Ni Li et al. formulated an OVA‐loaded chitosan nanoparticle with an average size of 258 ± 15 nm and a zeta potential of 27.6 ± 4.7 mV (**Figure** **4**a).^[44b]^ They found successful transdermal penetration in in vivo BALB/c mice, demonstrating vaccination. The anti‐OVA IgG profiles of mice were comparable to those injected with OVA and those treated topically with OVA‐loaded chitosan particles. Furthermore, with transdermal delivery of gp100‐loaded chitosan particles, mice exhibited a 33.81% reduction in tumor volume after 14 d, indicating successful delivery of gp100, while no noticeable decrease was observed in gp100 delivery without particles.^[44b]^ In the research by Xue Xie et al. carboxymethyl chitosan (CMC–chitosan) particles were used for the topical delivery system of bFGF with sonophoresis. The average size of bFGF‐loaded nanoparticles was measured as 81.9 nm using SEM images. FITC‐conjugated bFGF was used to observe the delivered bFGF, and ELISA was performed on skin lysate to quantify the amount. The delivered amount of bFGF with CMC‐chitosan nanoparticles without sonophoresis was observed to be 2.13 ± 0.21 μg mL^−1^. The maximum delivered amount of bFGF with sonophoresis in HR‐1 mice was measured as 7.59 ± 0.61 μg mL^−1^.^[44a]^

There is also protein skin delivery research using Pluronic F‐127, a well‐known polymer for its thermosensitive amphiphilic properties. Pluronic‐based nanocarriers were designed by Won Il Choi et al. to deliver insulin and BSA (Figure 4b).^[44e]^ Chitosan was conjugated to Pluronic F‐127 to increase skin permeability. The average size of protein‐loaded Pluronic nanoparticles was 60–61 nm, with an average zeta potential of −4.5–−4.3 mV. Chitosan conjugation on Pluronic displayed an increased nanoparticle size to 70–71 nm of average size and a positive zeta potential of 11.1–11.3 mV. Only the chitosan‐conjugated Pluronic F‐127 particle demonstrated transdermal delivery with bovine serum albumin (BSA)‐loaded in vitro human cadaver skin (back or thigh). However, Pluronic F‐127 nanoparticles alone also exhibited observable topical delivery and accumulation of BSA in the epidermal layer, as observed with fluorescence imaging of sectioned skin.^[44e]^

Kamran Tari et al. designed insulin‐loaded water‐soluble polypyrrole nanoparticles.^[^ ^47^ ^]^ The size of nanoparticles was 23 nm on average, and zeta potential was around – 38 mV. Although there was no efficient passive transdermal delivery, anodal iontophoresis (AIP) and cathodal iontophoresis (CIP) significantly enhanced insulin delivery flux, resulting in permeations of 520.0 ± 136.4 and 834.6 ± 218.9 μg cm^−2^ after 48 h, respectively.^[^ ^47^ ^]^

Therefore, chitosan is the only polymer demonstrated to passively deliver polymeric nanocarriers loaded with proteins without needing enhancers. Additionally, HA is a potential polymer that could be used, as reports already demonstrate protein delivery using HA conjugates. Although the properties of chitosan in opening the stratum corneum might be further studied and utilized, it is controversial due to the potential irritation and toxicity associated with its positive functional groups.^[^ ^48^ ^]^ Therefore, careful consideration should be taken when selecting chitosan as a drug delivery carrier.

### 2.3 Inorganic Nanoparticle‐Based Delivery Systems

Due to their high chemical and mechanical stability, inorganic nanoparticles are explored for topical and transdermal delivery (**Table** **3**).^[^ ^49^ ^]^ Moreover, easy modification of their chemical and physical properties makes inorganic nanoparticles suitable for transdermal delivery and demonstrates the potential for additional functions such as bioimaging,^[^ ^50^ ^]^ UV protection,^[^ ^51^ ^]^ and antibacterial activity.^[^ ^52^ ^]^ Silver nanoparticles are especially well known for their antibacterial properties, high surface area, facile surface modification capabilities, and cell penetration efficiency. Jun Tian et al. topically delivered silver nanoparticles to wound sites and demonstrated their antibacterial effects, promoting wound healing compared to mice without treatment.^[^ ^53^ ^]^ Moreover, gold nanoparticles also show potential as skin delivery carriers since they have a large surface area and easy functionalization properties. Their electrical and magnetic chemical properties also make them easy to combine with other enhancers, and their plasmonic properties are utilized for imaging, sensing, or photothermal applications.^[^ ^54^ ^]^ The penetration mechanism of gold nanoparticles was studied through simulation, proposing that they induce undulation and create vacancies inside the bilayer. After penetration, the lipid layer self‐heals, resulting in no disruption of the lipid layers.^[^ ^55^ ^]^ Although silver and gold nanoparticles are less studied for protein delivery due to controversies surrounding their toxicity and solubility limitations, some research on gold nanoparticles has been investigated for protein transdermal delivery due to their relatively easier tailoring. Therefore, studies have been conducted on transdermal delivery of GF, OVA, antibodies, and insulin using gold nanoparticles.^[^ ^56^ ^]^

**Table 3:** Inorganic nanoparticles‐assisted protein transdermal and topical delivery.

Nanoparticle type	Subtype	Characteristic (size, zeta potential)	Protein	Enhancer	Results	Reference
Inorganic nanoparticle	Gold nanoparticle	3.22 ± 0.09 nm	OVA	–	Transdermal vaccination was observed in in vivo model. The averaged titer of OVA‐speciﬁc IgG in the OVA‐conjugated gold nanoparticle group was 466 times higher than the OVA group.	[56a]
		–				
	Gold nanoparticle	15.1 nm ±0.1 nm + 8–10 nm	VEGF	–	Higher penetration was observed with gold nanoparticles and the system induced angiogenesis with delivered VEGF	[56b]
		–				
	Gold nanorod (hyaluronate‐conjugated)	15.69 nm and 91.28 nm	DR5 antibody	–	Gold nanorod–hyaluronate complex was used to penetrate the DR5 antibody and target cancer. Photoacoustic imaging clearly visualized the eﬀective transdermal delivery of HA‐AuNR/DR5 Ab complex, penetrating the skin barrier signiﬁcantly compared to DR5 Ab alone.	[56c]
		−4.20 ± 1.20 mV				
	Hollow copper sulﬁde nanoparticle	≈55 nm	hGH	Photothermal ablation	Nanosecond‐pulsed NIR laser application local ablation of stratum corneum. With a 2.6 W cm^−2^ laser, a 2.9 ± 0.3 cm h^−1^ permeation rate was observed in vitro, which was 6.3‐fold higher than the chemical enhancer Azone.	[59a]
		–				
	MSN (gluconamide, ZnO dots, and phenylboronic acids functionalized)	100–120 nm	Insulin	Microneedle	A capping bond was detached in high blood glucose levels, and insulin was released from pores. MSN carrier with microneedle‐treated mice showed a 3.5 h longer time of blood glucose regulation (<200 mg dL^−1^) than insulin treated only.	[58a]
		–				
	MSN (4‐(imidazoyl carbamate) phenylboronic acid and CD complex functionalized)	192 nm	Insulin, GOx	Microneedle	In the presence of glucose and oxygen, GOx generates H_2_O_2_, and ICBE molecules are degraded, thus resulting in the destruction of host–guest complexation and subsequent release of the insulin. The BGLs can be retained for around 4.5 h in the normoglycemic range (under 200 mg dL^−1^) compared with only 2 h for the subcutaneously injected group	[58c]
		−13.7 mV				
	Mesoporous bioactive glasses (ZnO QDs coated)	253 nm	GOx, insulin, catalase	Microneedle	GOx and catalase lower the local pH in high glucose levels, and ZnO QDs degrade at the low pH and the insulin releases. It maintained its lower level (<200 mg dL^−1^) for 6 h.	[58b]
		14.8 mV				

**Figure 5:**
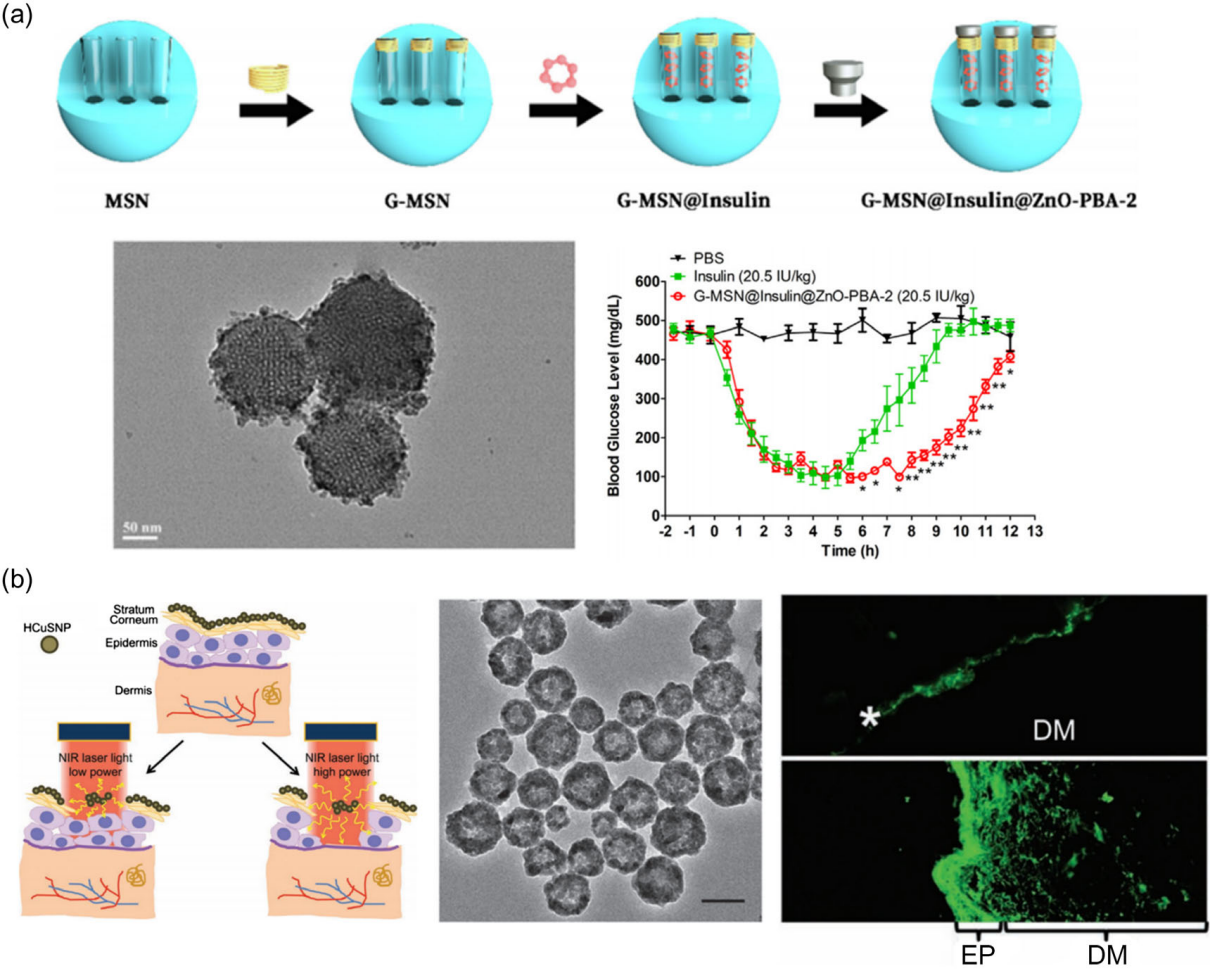
Inorganic nanoparticle‐assisted protein delivery. a) Insulin was loaded into MSNs with a glucose‐sensitive linker. At high glucose levels, the connection cleaved, releasing insulin and displaying excellent glycemic control. Reproduced with Permission.^[58a]^ Copyright 2021, Elsevier. b) The photothermal effects of hollow copper sulfide nanoparticles were used for transdermal delivery. The photothermal effect ablated the stratum corneum, thereby increasing the skin permeability to human growth hormone. Reproduced with Permission.^[59a]^ Copyright 2012, Wiley.

Moreover, silica nanoparticles are utilized for their excellent mechanical strength and easy morphological changes. Specifically, mesoporous silica nanoparticles (MSN) are known to play significant roles in transdermal or topical delivery, facilitating skin adhesiveness and skin retention and providing a large drug reservoir.^[^ ^57^ ^]^ MSNs show significant advantages in protein encapsulation due to their high porosity and the easy adhesion of proteins onto porous areas.^[^ ^58^ ^]^ In particular, insulins were usually encapsulated in MSN. Yun Fu et al. developed insulin‐encapsulated MSN with phenylboronic acid‐grafted zinc oxide dots to prevent insulin from detachment (**Figure** **5**a).^[58a]^ Phenylboronic acid and gluconamide detached in high glucose concentration conditions, releasing insulin. They exhibited over 20% insulin release within 20 h at 450 mg dL^−1^, and controlled release was also observed at different glucose concentrations, indicating pulsatile release. Particle delivery was assisted with microneedles, and controlled glucose levels were observed in vivo. Although MSN showed high insulin loading efficiency, there have been no studies using MSN for transdermal insulin delivery without noninvasive enhancers. Minimally damaging the skin with microneedle patches loaded with insulin‐loaded MSN significantly impacted glucose levels. However, there is potential for noninvasive delivery of MSN, as demonstrated by Zhiyuan Zhao et al., which used deep eutectic solvents (DESs) (ILs) to enhance the transdermal delivery of MSN noninvasively.^[^ ^57^ ^]^ The particles of 120 μm showed penetration of over 50 μm into the epidermal and dermal layers of porcine ear skin when delivered topically. Using ILs, the penetration depth reached up to 100 μm. When the ILs were conjugated on the surface of MSN, they penetrated the skin and facilitated transdermal delivery. This study demonstrates the potential for protein transdermal delivery using DES‐MSN.

Other inorganic particles using metallic compounds (such as copper or metal–organic framework [MOF]) can also be used as carriers. Their mechanical and thermodynamic properties, such as stiffness, adhesiveness, photothermal effect, and photodynamic effect, can also be used to enhance the protein delivery efficacy.^[^ ^59^ ^]^ Samy Ramadan et al. developed hollow copper sulfide (CuS) nanoparticles topically delivering hGF (Figure 5b).^[59a]^ Utilizing the photothermal effect of CuS, the stratum corneum can be penetrated to deliver the nanoparticles with the protein. The particle size was made to be 55 nm in diameter, which showed a photothermal effect, increasing the temperature up to 50 °C with 2.6 W cm^−2^ laser (900 nm) irradiation in 5 s. Then, it was loaded onto hGH hydrogel, which was applied to nude mice. The photothermal effect of CuS nanoparticles successfully ablated the stratum corneum, resulting in skin permeability of 2.9 ± 0.3 10^−3^ cm h^−1^, which was 6.3‐fold higher than that of the chemical enhancer Azone.

Although inorganic materials have many advantages in biological and clinical applications due to their various properties, there are few studies reported on protein skin delivery. Their toxicity, low solubility, and low loading efficiency are significant hurdles. MSNs have emerged as a way to increase protein loading efficiency. However, their low penetration limits application. Therefore, MSN‐guided protein delivery has been used with microneedles. To avoid the use of invasive enhancers, increasing the lipophilicity or changing the morphology of particles can improve transdermal efficiency. This is more easily achieved with inorganic particles compared to other types due to their ease of tailoring.

### 2.4 Carbon Nanotube‐Based Delivery Systems

**Figure 6:**
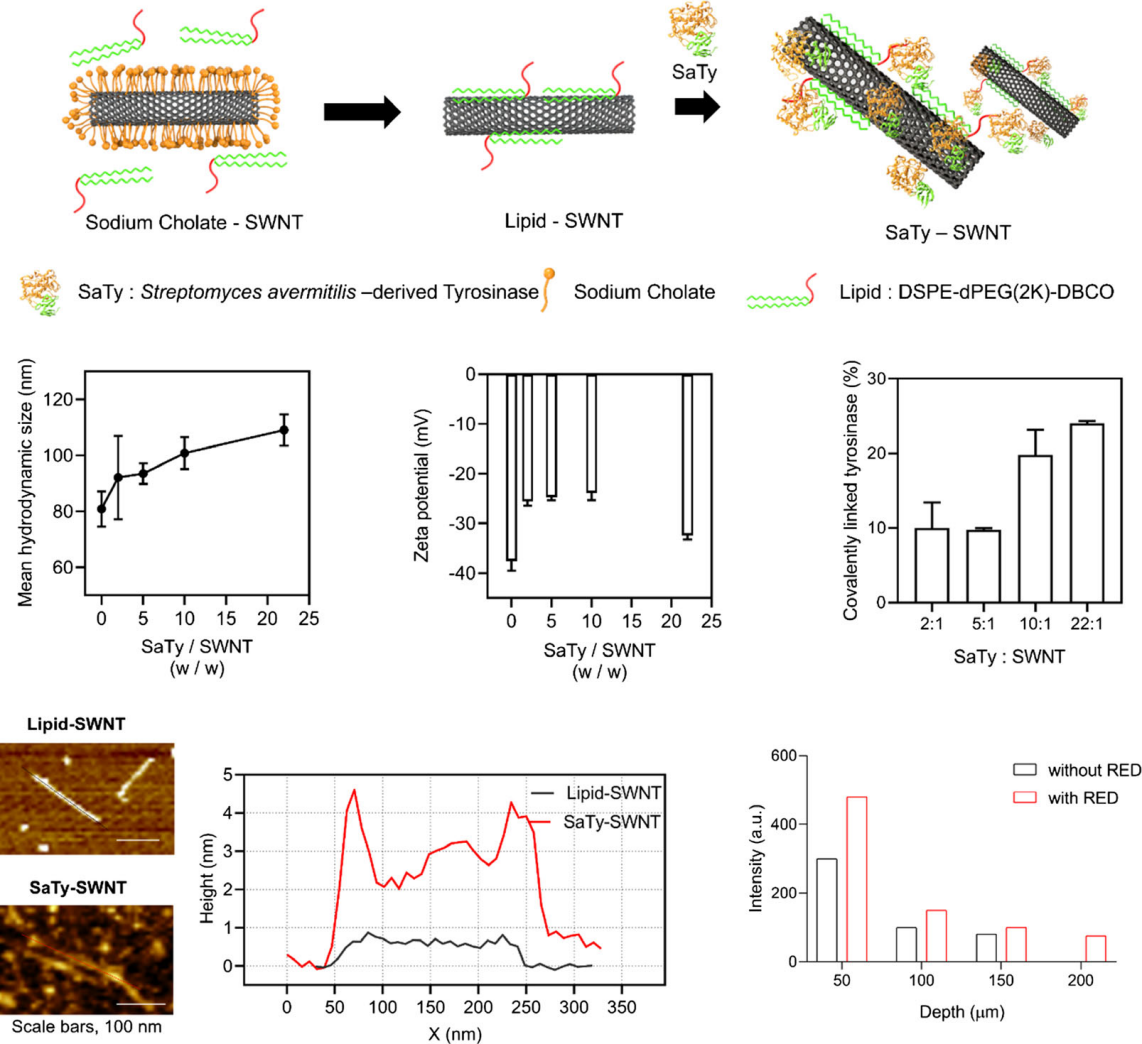
SWNT was utilized to deliver the enzyme tyrosinase topically. Loading efficiency was confirmed while maintaining the size and the zeta potential of the nanoparticle. The lipid was coated onto SWNT to increase lipophilicity and skin permeability. Reproduced with Permission.^[^ ^61^ ^]^ Copyright 2023, ACS Publications.

Carbon nanotubes are mechanically strong and stable materials, classified into single‐walled, double‐walled, and multiwalled carbon nanotubes. Single‐walled carbon nanotubes (SWNTs) have a high aspect ratio and large surface area. It can be easily functionalized, which allows it to be adjusted to the materials or target tissue. Moreover, their needle‐like structure is known for penetrating biological barriers and exhibiting tissue‐penetrating effects.^[^ ^60^ ^]^ Therefore, some small drugs have been loaded for transdermal or topical delivery using carbon nanotubes. However, protein delivery with carbon nanotubes has not been extensively studied. Jungehyeon Ko et al. developed SWNT derivatives showing stratum corneum‐penetrating effects with an average particle size ranging from 65.6 ± 5.2 to 87.2 ± 13.2 nm (**Figure** **6**, **Table** **4**).^[^ ^61^ ^]^ Moreover, they showed that tyrosinase‐loaded SWNTs (SaTy‐SWNT), with a size of 109.1 ± 5.6 nm, successfully penetrated to a depth of 150 μm without any enhancers. To increase the penetration ability, lipophilicity was applied to the SWNT by coating it with lipid. Additionally, carbon nanotubes have an inherent G‐band at 1590 cm^−1^ in Raman spectra, which is used to determine the location of penetrating protein and carbon nanotube conjugates. This provides an advantage over other nanocarriers that require additional agents, such as fluorescent dyes, to be detected inside tissues or organs. Thus, Junghyeon Ko et al. used Raman spectra to observe penetration depth and biodistribution, helping to confirm the localization of tyrosinase and SWNTs within the dermal layer, without dispersal into the systemic circulation.

**Table 4:** Carbon nanotube‐assisted protein topical delivery.

Nanoparticle type	Subtype	Characteristic (size, zeta potential)	Protein	Enhancer	Results	Reference
Carbon nanotube	SWNT (Lipid coated)	109.1 ± 5.6 nm	Tyrosinase (SaTy)	Iontophoresis (RED)	Tyrosinase‐loaded carbon nanotube (SaTy‐SWNT) penetrated ex vivo porcine skin ≈150 μm passively and 300 μm with RED battery, exhibiting melanin synthesis. With in vivo BALB/c mice, UV protection by melanin synthesis and wrinkle alleviation by dermal crosslinking by delivered SaTy‐SWNT were observed.	[61]
		−32.4 ± 0.81 mV				

### 2.5 Strategies to Enhance Nanoparticle Physical Properties for Effective Skin Penetration

Although most studies have focused on selecting types of nanoparticles for protein transdermal carriers, the properties determining tissue penetration ability are more than that, including surface charge, morphology, and size. Therefore, future studies could be improved by developing upon recent studies and considering the findings of previous research conducted on small drugs.

#### 2.5.1 Surface Charge

**Figure 7:**
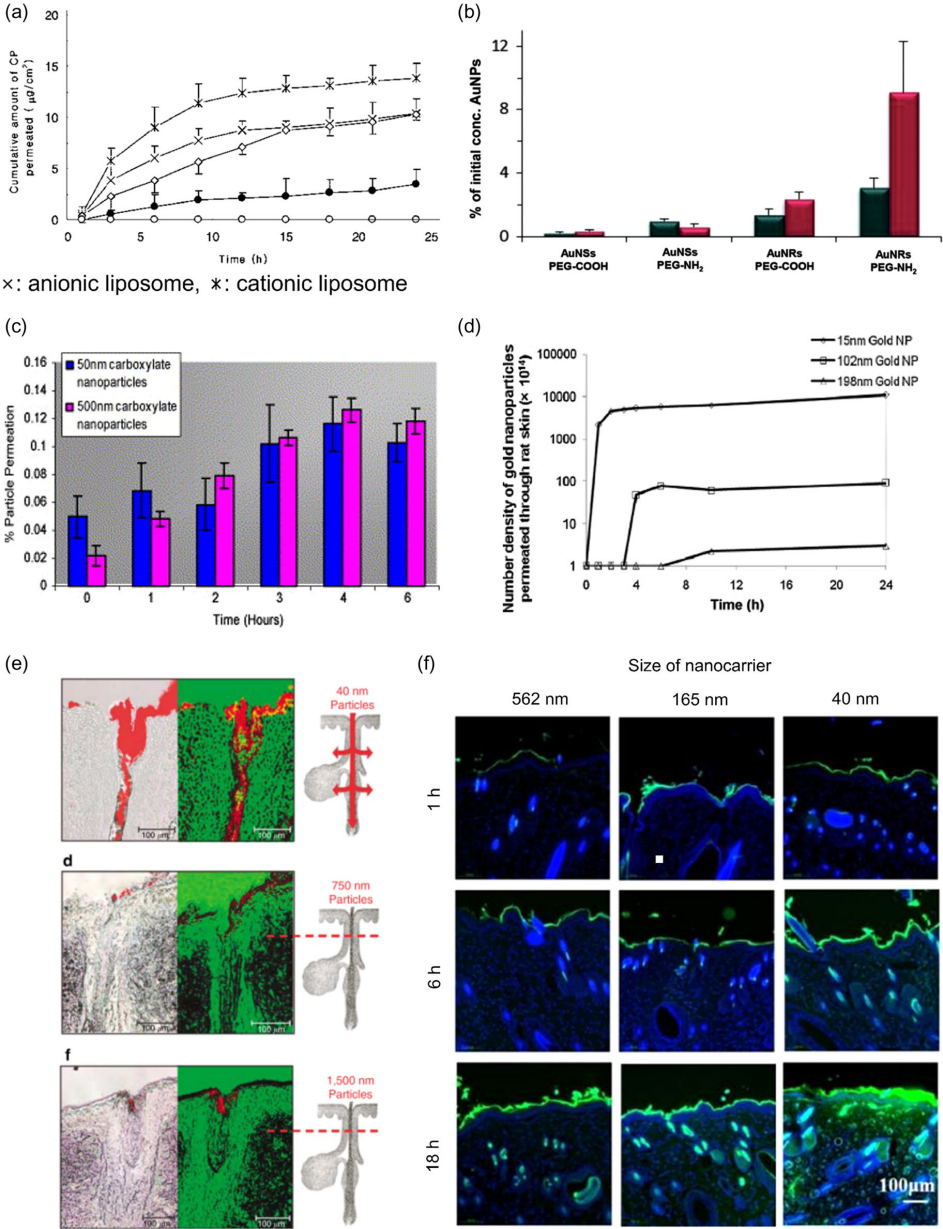
a) The skin penetration abilities of anionic and cationic liposomes were compared to assess the impact of surface charge on transdermal delivery. Reproduced with Permission.^[64a]^ Copyright 2009, Springer. b) Gold nanoparticles with different shapes and surface charges were compared. Reproduced with Permission.^[64b]^ Copyright 2014, Wiley. c) The skin permeability of 50 and 500 nm carboxylated nanoparticles was compared. Reproduced with Permission.^[^ ^65^ ^]^ Copyright 2003, Elsevier. d) Skin permeability was compared between gold nanoparticles of different sizes (15, 102, and 198 nm). Reproduced with Permission.^[71a]^ Copyright 2008, Elsevier. e) The topical delivery ability at skin hair follicles was compared using polystyrene nanoparticles of various sizes (40, 750, and 1500 nm). Reproduced with Permission.^[^ ^69^ ^]^ Copyright 2006, Elsevier. f) The degree of penetration over time was compared for silk nanoparticles of different sizes (40, 165, and 562 nm). Reproduced with Permission.^[71b]^ Copyright 2023, ACS Publications.

The skin carries an overall negative charge, with an average fixed charge density of −2.5 mM, which is calculated based on the content of glycosaminoglycan chains.^[^ ^62^ ^]^ The skin has an isoelectric point (pI) of 4–5 and a pH range of 4–7. This pH gradually increases toward the dermis, indicating that the negative charge within the skin tissue becomes stronger.^[^ ^63^ ^]^ The surface charge of nanoparticles used for effective transdermal delivery systems is controversial, considering the negative charge of the skin. Much of the research prefers cationic nanoparticles, considering the electrostatic attractive force between the skin surface and the nanoparticles, potentially facilitating initial penetration (**Figure** **7**a,b).[^48^, ^64^] However, another study by A.K. Kohli et al. indicates that positively charged particles do not penetrate the skin, whereas negatively charged nanoparticles exhibit higher penetration. They suggest that negatively charged particles permeate the skin by creating provisional channels through repulsive forces between the negatively charged lipids within the skin and nanoparticles, facilitating their movement through the epidermal and dermal layers, even for large particles up to 500 nm in size (Figure 7c).^[^ ^65^ ^]^ Overall, nanoparticles with a positive charge on the surface demonstrate easier penetration into the skin layer due to electrostatic attraction. After the nanoparticles penetrate the stratum corneum, their further penetration is influenced by both attraction and repulsion. However, surface charge is not the only characteristic affecting attraction or repulsion. Researchers should also consider π‐π interactions, hydrogen bonding, or other noncovalent interactions between tissue and nanoparticles when selecting the nanoparticle.^[^ ^54^, ^57^, ^66^ ^]^ Moreover, studies indicate that positive functional groups, such as amino groups, possess skin‐irritating and cell‐damaging properties.[^48^, ^67^] Materials should be cautiously selected when applying a charged surface.

#### 2.5.2 Size

Although researchers are targeting the narrow intercellular route, most studies on the transdermal or topical delivery of nanoparticles have shown follicular penetration (Figure 7d–f).^[^ ^68^ ^]^ This follicular penetration was even observed for particles larger than 700 nm (e.g., 750 nm, 1500 nm), which tend to aggregate at the infundibulum (Figure 7e). Such aggregation makes them unsuitable for dermal topical delivery and transdermal delivery.^[^ ^69^ ^]^ Given the brick‐and‐lamellar structure of packed corneocytes and the lipid matrix,^[^ ^70^ ^]^ selecting the proper size of the nanoparticle is crucial to penetrating through the lamellar structure. Research on nanoparticles smaller than 250 nm has demonstrated that they can exhibit skin permeability, indicating a potential for transdermal effects (Table 1, 2, 3, 4). It is easy to understand that as the size of the nanoparticles decreases, skin permeability increases, resulting in higher transdermal flux (Figure 7d,f).^[^ ^71^ ^]^ However, research shows that particles sized at 500 nm can penetrate the skin, a result explained by the electrostatic repulsive effect of the lipid layer within the skin.^[^ ^65^ ^]^ Therefore, controlling not only the size but also the charge, morphology, and materials used is essential.

#### 2.5.3 Morphology (Shape)

The shape or morphology of the particle is one of the parameters that controls skin penetration ability. Studies have shown that the shape of the particle affects its ability to penetrate or interact with cellular uptake.^[^ ^72^ ^]^ However, limited research has been conducted on the impact of particle shape on their ability to penetrate skin and interact with skin tissue. Research investigating shape‐dependent skin penetration, particularly with gold nanoparticles, has found that rod‐ or star‐shaped particles exhibit higher penetration than sphere type (Figure 7b).^[^ ^54^ ^]^ Therefore, other rod‐like materials such as carbon nanotubes,^[^ ^61^ ^]^ nanodisc,^[^ ^34^ ^]^ or nanorods^[56d]^ have been studied for promising skin drug delivery systems. Although star‐shaped nanocarriers have not been extensively studied as drug carriers themselves, they have been investigated for their role as enhancers. Andrew R. Tadros et al. developed star‐shaped microparticles that painlessly disrupt the stratum corneum, facilitating efficient drug delivery through or into the dermal layer.^[^ ^73^ ^]^ They observed topical delivery using dextrans of different molecular weights and successfully delivered vaccines with molecular weights exceeding 100 kDa.

## 3 Enhancers with Nanoparticles for Effective Protein Delivery

Although researchers are trying to achieve protein‐loaded nanoparticle delivery without additional enhancers, efficiency remains low with nanoparticles alone. Since nanoparticles are larger than proteins, some nanocarriers are less effective in protein delivery than simply delivering the proteins passively. Therefore, various chemical and physical enhancers are used to increase the delivery depth and improve targeting properties.^[11a]^ Moreover, by modifying the surface or using additional materials, we can design various types of nanocarriers, tailoring them to exhibit specific properties such as electromagnetic responsiveness, pH sensitivity, and charge reversibility. These selected properties can be matched with the desired functions of enhancers, optimizing the delivery system effectively. However, most delivery methods have not shown sufficient results in penetrating the dermal layer and delivering materials systemically. Although microneedles or injections are the most precise methods for drug delivery, damage to skin tissue is inevitable. Therefore, this review will discuss noninvasive enhancers, increasing the potential of nanoparticles to deliver proteins effectively.

### 3.1 Electrically Assisted Penetration

Electrically assisted penetration enhancement is a well‐known system for facilitating transdermal or topical delivery due to its noninvasiveness and controllability. When the enhancer charge sign is equal to that of the charged nanoparticles, electrostatic repulsive force facilitates delivery. Moreover, by controlling the duration, both the depth and amount of delivery can be regulated. The electroporation and iontophoresis systems are mainly used for electrostatic repulsive delivery. The electroporation system delivers drugs using an instantaneous high‐voltage pulse (usually more than 50 V).^[^ ^74^ ^]^ Iontophoresis is the method that creates a charge or voltage gradient within skin tissue by generating a weak current, making the drug move toward the opposite charge gradient.^[^ ^75^ ^]^ Designing an iontophoretic device should be done with care to avoid damaging skin tissue while simultaneously delivering the drug safely and accurately. The power or current density should be optimized to ensure efficient delivery while minimizing damage to the skin or proteins. Moreover, by controlling the surface charge of the nanoparticles, the effectiveness of the iontophoresis system can be maximized.[^44^, ^76^] Therefore, many studies have designed charged nanoparticles to enhance the effect of iontophoresis for delivering various proteins, including insulin, enzymes, or OVA.[^27^, ^44^, ^47^, ^61^]

Kamran Tari et al. studied the delivery of insulin, which was loaded onto water‐soluble polypyrrole nanoparticles.^[^ ^47^ ^]^ The nanoparticles exhibited a zeta potential of −38 mV, and the iontophoresis was conducted at 0.13 mA cm^−^^2^. Passive penetration exhibited a rate of 0.36 ± 0.09 μg cm^−^^2^ h^−1^. With AIP, which employs an attractive force, a slight increase in transdermal flux was observed, achieving a maximum penetration rate of 9.59 ± 2.65 μg cm^−^^2^ h^−1^. On the other hand, CIP, utilizing a repulsive force, delivered insulin more effectively and rapidly, with a transdermal flux of 0.36 ± 2.65 μg cm^−^^2^ h^−1^.

**Figure 8:**
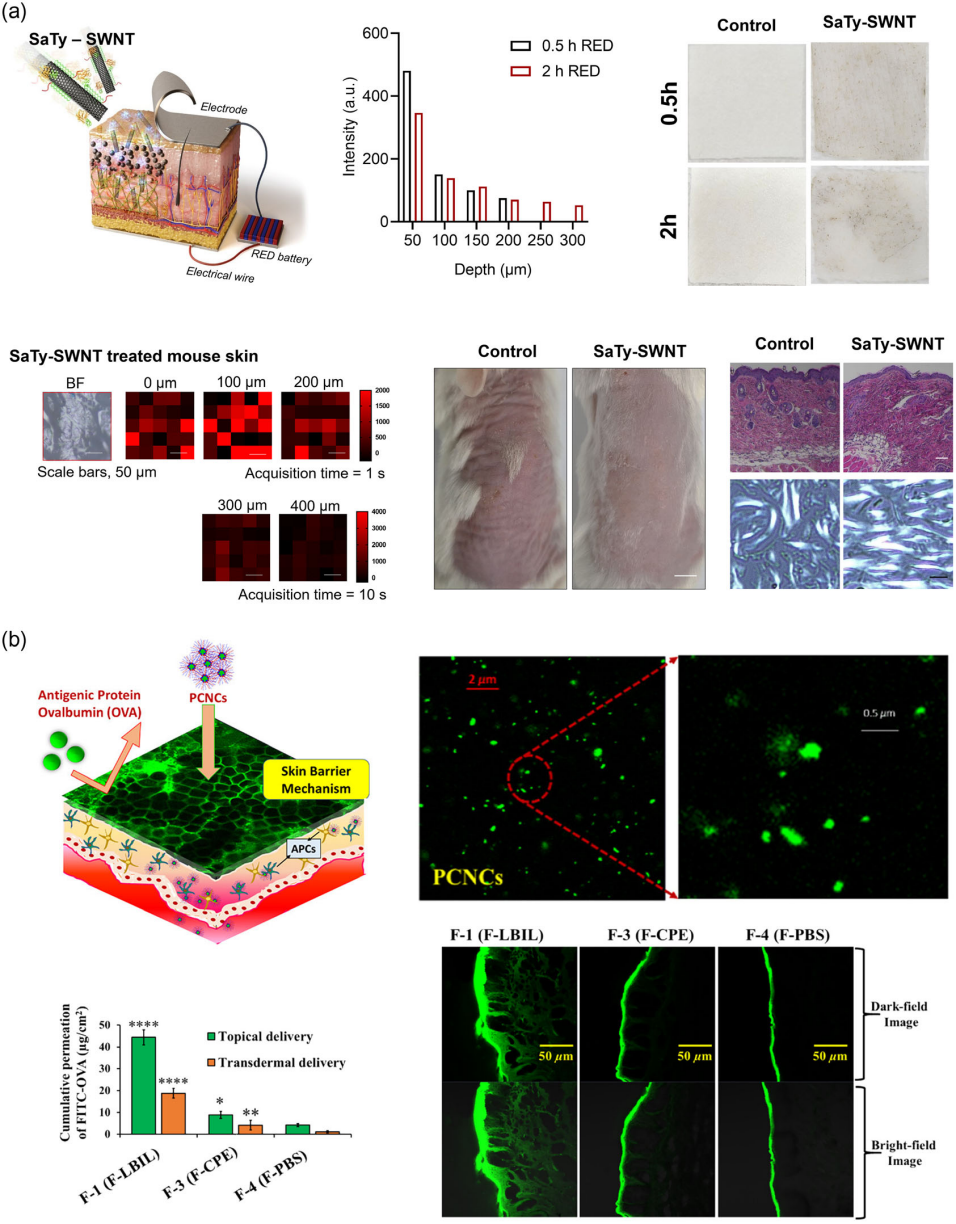
a) Tyrosinase topical delivery mediated by SWNT was enhanced and controlled using a RED battery. The penetration depth increased, and the amount of synthesized melanin was enhanced with the application time of the RED battery. The delivered tyrosinase increased dermal density, which reduced photoinduced damage and wrinkles. Reproduced with Permission.^[^ ^61^ ^]^ Copyright 2023, ACS Publications. b) OVA‐loaded nanocarrier skin delivery was enhanced by ILs. Reproduced with Permission.^[80b]^ Copyright 2022, ACS Publications.

Junghyeon Ko et al. developed negatively charged SaTy‐SWNTs (−32.4 ± 0.8 mV) and electrostatically repulsed the particle using a reverse electrodialysis (RED) battery system (**Figure** **8**a).^[^ ^61^ ^]^ The RED system increased the maximum delivery depth of tyrosinase from 150 to 300 μm with a 3 h application. Moreover, by controlling the duration of delivery, the enzyme reached a depth of 200 μm with a 0.5 h application and 300 μm with a 2 h application. Their delivery system was selected to target the dermal extracellular matrix. Thus, topical delivery control was observed with the RED system.

Daniela Bernardi et al. transdermally delivered OVA‐loaded liposomes incorporated with silver nanoparticles.^[27f]^ This nanosystem exhibited a zeta potential of −14 ± 1 mV. Therefore, cathodic iontophoresis was selected to enhance the transdermal efficacy. With iontophoresis and the nanosystem, in pig ear skin, the delivery amount exceeded 4 mg cm^−^^2^, while the passive delivery of OVA was less than 2 mg cm^−^^2^ on average in the epidermis.

### 3.2 Ionic Liquid and Deep Eutectic Solvents (DESs)

ILs and DESs have recently emerged as penetration enhancement systems, offering benefits such as easy application and good stability to improve protein delivery. An IL is a salt composed of an organic cation and either an organic or inorganic anion, characterized by a melting point lower than 100 °C.^[^ ^77^ ^]^ DES is composed of both charged and neutral species, mixtures of a variety of Lewis or Brønsted acids and bases.^[^ ^78^ ^]^ Both IL and DES change the structure of the stratum corneum, disturbing the alkyl chains of stratum corneum lipids and increasing fluidity.^[^ ^79^ ^]^ The separation of lipids increases the hydrophilic regions, enhancing penetration efficacy. Moreover, since IL and DES are ionically designed, they enhance drug solubility by enveloping the drugs similarly to micelles. These properties increase nanoparticle delivery by improving both the solubility of the particles and their interaction with skin tissue.^[^ ^57^ ^]^ Utilizing the IL or DES system, the delivery of proteins such as insulin or OVA through the skin has been studied.^[^ ^80^ ^]^

Zhiyuan Zhao et al. screened combinations of amino acids and citric acid to identify the best DES for delivering MSN. They found that DES‐crosslinked particles achieved maximum penetration of up to 400 μm, and the transdermal flux was ≈0.4 mg cm^−2^ h^−1^ in rat skin delivery.^[^ ^57^ ^]^ Although they didn't load any protein drugs, this research demonstrates the potential of the system for applications similar to previous research on MSNs containing insulin.

Shihab Uddin et al. fabricated a solid–oil nanodispersion system to deliver OVA and ILs were used to assist in increasing penetration ability (Figure 8b).^[80b]^ The transdermal flux was 7.6 ± 0.6 μg m^−^^2^ h^−1^ for the OVA‐loaded carrier assisted by IL, while the carrier without IL exhibited only 0.3 ± 0.1 μg m^−^^2^ h^−1^. Moreover, in mouse ear skin, highly accumulated OVA in the epidermis was observed with nanocarrier and without IL, with hardly any penetrated OVA observable in the dermis. In contrast, OVA presence extended down to the dermal region in the system delivered by the IL‐assisted nanocarrier.

### 3.3 Other Noninvasive Delivery System

As many noninvasive delivery systems, such as sonophoresis and magnetic guidance, have been studied for small drug and nanoparticle delivery, their application to large macromolecules or proteins is also being explored.

Sonotophoresis is a delivery method that uses ultrasound to guide drugs. This method induces acoustic cavitation, generating waves that change lipid structures to create diffusion channels. Moreover, ultrasound energy increases skin temperature, enhancing diffusion flux. Xue Xie et al. fabricated chitosan nanoparticles encapsulating bFGF.^[44a]^ Ultrasound was applied with a transducer. With a 120 mA current generating ≈75 kPa of acoustic pressure, it delivered bFGF‐loaded chitosan nanoparticles topically, achieving a concentration of about 7.57 ± 0.71 μg mL^−1^. This is significantly higher than the concentration achieved by sonophoretic delivery of bFGF without the chitosan nanocarrier, which was 5.1 ± 0.48 μg mL^−1^.

### 3.4 Minimally Invasive System: Microneedle and Nanoneedle

Microneedles utilize microsized needles, ranging from 10 to 2000 μm in height, to minimally damage the skin and deliver drugs. Although there are concerns about tissue damage when using microneedles, they cause minimal harm to the skin, are painless, and deliver drugs more effectively than other enhancement methods. The design of the microneedle scaffolds allows for controlled drug release profiles.^[^ ^81^ ^]^ Thus, microneedles, in combination with nanoparticles, can stabilize proteins and regulate their delivery by enhancing stratum corneum penetration. This approach has successfully facilitated the controlled release of proteins such as insulin or OVA through nanoparticle and microneedle systems.[^44^, ^46^, ^58^, ^59^, ^82^]

Nak Won Kim et al. designed a Pluronic F127‐based polymeric nanoparticle to encapsulate both hydrophobic and hydrophilic materials, including a Toll‐like receptor 7/8 agonist (R848) and OVA.^[82b]^ The nanoparticles were fabricated with sizes ranging from 30 to 40 nm, and their delivery was enhanced by dissolving microneedles. The microneedle‐assisted delivery of nanoparticles and proteins successfully immunized C57BL/6 mice, leading to effective tumor prevention.

Xiao‐Xi Yang et al. designed a MOF‐based insulin and glucose oxidase delivery system, which was enhanced by a PVA‐based microneedle of 650 μm in height.^[59b]^ The MOF particle, larger than 500 nm, faced challenges in penetrating the skin by itself. Glucose oxidase acts as a glucose‐responsive factor, catalyzing glucose into gluconic acid and releasing H_2_O_2_. This process lowers the local pH and triggers the release of insulin. Although the in vivo confirmation of insulin release or response to glucose was not provided, in vitro studies demonstrated controlled insulin release dependent on glucose concentration. Ex vivo experiments showed successful skin penetration in porcine models.

Jicheng Yu et al. reported on a hypoxia‐sensitive hyaluronic acid‐based nanocarrier with an average size of 118 nm, designed to deliver insulin in a diabetic model.^[82c]^ Under high glucose levels, local oxygen is consumed, creating a hypoxic environment. This environment reduces nitroimidazole to aminoimidazole, causing the self‐assembled nanocarrier to dissociate and release the encapsulated insulin. Microneedles were used to enhance the delivery of the nanoparticles. They confirmed a decrease in glucose levels and observed a normoglycemic state (<200 mg dL^−1^) for up to 4 h.

Since microneedles have raised concerns regarding tissue damage, nanoneedles have been studied for their ability to noninvasively contact tissues or cells.^[^ ^83^ ^]^ Nanoneedles or nanoprobes are emerging techniques with a high aspect ratio designed to interact with biointerfaces. They have been suggested to create biointerfaces at the cellular scale, interacting with cells for applications such as biosensing, delivery, and electrical stimulation.^[^ ^84^ ^]^ Some studies have also demonstrated the use of nanoneedles for topical skin delivery or transdermal delivery as a noninvasive delivery system.^[^ ^85^ ^]^ However, nanoneedles use shorter needles with smaller diameter, making them less applicable than microneedles for systemic transdermal delivery systems. Therefore, only the small scales of drugs or topical delivery have been studied. This drawback can be addressed by using nanoparticles as carriers or enhancers to increase protein penetration depth and stability.^[^ ^86^ ^]^

## 4 Future Perspectives of Nanoparticles for Protein Transdermal Delivery

Drug topical and transdermal delivery, particularly for small drugs, has been extensively studied in association with nanoparticles and enhancers. However, due to the large molecular size of proteins, which makes their delivery through the dense layer of skin challenging, there have been significantly fewer studies on protein delivery. It is essential to determine the molecular weight of proteins that pose a challenge for skin delivery, even when using nanoparticles and enhancers.

The size, morphology, and surface charge of nanoparticles should be considered simultaneously. Particularly, the types of nanoparticles or loaded proteins might determine the system's elasticity or flexibility, affecting its ability to penetrate the dense stratum corneum. However, there are not many studies on the correlation between loaded protein types and their mechanisms of affecting delivery efficacy, such as flexibility or elasticity. A detailed comprehension of the mechanism could help determine the design of nanoparticles according to the types and amounts of protein to be delivered.

Although further research is required, various studies have showcased the remarkable capabilities of nanotechnology to enhance protein stability and ensure precise delivery. Moreover, these improvements in nanotechnology for protein carrier or skin barrier penetration could be helpful for other topical barrier‐penetrating applications such as the eyes and mucosa (gastrointestinal, genital, oral, nasal, etc.).^[^ ^87^ ^]^ These cutting‐edge technologies offer a noninvasive method for delivering proteins, with potential applications in critical medical areas such as managing glucose levels in diabetic patients. Integrating nanotechnology in transdermal protein delivery systems holds potential in future medical treatments and methodologies.

## Conflict of Interest

The authors declare no conflict of interest.
